# The Causes of Insulin Resistance in Type 1 Diabetes Mellitus: Is There a Place for Quaternary Prevention?

**DOI:** 10.3390/ijerph17228651

**Published:** 2020-11-21

**Authors:** Marta Wolosowicz, Bartlomiej Lukaszuk, Adrian Chabowski

**Affiliations:** Department of Physiology, Medical University of Bialystok, Mickiewicza 2c Str., 15-222 Bialystok, Poland; bartlomiej.lukaszuk@umb.edu.pl (B.L.); adrian@umb.edu.pl (A.C.)

**Keywords:** type 1 diabetes, insulin resistance, quaternary prevention, metformin, screening, diagnosis, risk factors, multidimensional approach

## Abstract

Diabetes mellitus was the first non-communicable disease that was recognized by the United Nations as a 21st-century pandemic problem. Recent scientific reports suggest that people with type 1 diabetes mellitus also develop insulin resistance, which is generally considered to be a distinctive feature of type 2 diabetes mellitus. The causes of insulin resistance in type 1 diabetes mellitus were explored, but there was a lack of publications that connected the risk factors of insulin resistance in type 1 diabetes mellitus with the proposition of repair mechanisms that are offered by quaternary prevention. Toward this end, the present review is an attempt to combine the previous reports on the causes of insulin resistance in type 1 diabetes mellitus and a brief review of quaternary prevention. The destructive effect of insulin resistance on many physiological processes that predisposes the individual to chronic diabetes complications creates an urgent need to introduce effective therapeutic methods for preventing the development and progression of this pathology.

## 1. Introduction

Since the beginning of civilization, humanity has struggled with many epidemics of infectious diseases. After they had been defeated, mankind faced another problem of epidemic or rather pandemic proportions, i.e., non-communicable diseases. Currently, non-communicable diseases are the main cause of morbidity and mortality around the world. Diabetes mellitus (DM) was the first non-communicable disease that was recognized by the United Nations as a 21st-century pandemic problem [1]. DM is a metabolic disease, characterized by an inappropriately elevated blood glucose level. DM is divided into several types that are based on the pathogenesis of the disease. The two most common forms of the condition are type 1 and type 2 DM. Table 1 provides a concise comparison of these two types of DM.

Type 1 diabetes mellitus (T1DM), which was previously known as insulin-dependent, or childhood diabetes, is characterized by a deficiency in insulin production and, thus, requires constant administration of exogenous insulin. Recent scientific reports suggest that people with T1DM also develop insulin resistance (IR), a phenomenon that is generally considered to be a distinctive feature of T2DM. Therefore, this review will focus on the pathogenesis of IR in T1DM, as well as its quaternary prevention and treatment [2].

Interestingly, the majority of people with diagnosed DM live in the Western Pacific (131 million) and South-East Asian Regions (96 million) [3]. However, the population of people with DM in the European (64 million) and American (62 million) Regions is also quite large [3]. This spatial distribution may be due to the increase in the number of overweight and obese people in those regions [4]. According to the WHO report, nowadays, one in three adults is overweight, and more than one in 10 is obese [3]. In 2014, it was estimated that 422 million adults (8.5% of the world adult population) live with DM, when compared to 108 million in 1980 (4.7% of the world adult population) [3]. DM is the seventh leading cause of death and a major cause of costly and debilitating complications, such as heart attack, stroke, kidney failure, blindness, and lower-limb amputation.

## 2. Insulin

Insulin is a highly-anabolic endocrine hormone that is secreted by β-cells of the Islets of Langerhans located in the pancreas. It plays a wide spectrum of metabotropic roles, e.g., it stimulates transmembrane transport of glucose, drives the glycolysis process, triggers glycogen synthesis, and abolishes its breakdown [5]. Moreover, insulin silences lipolytic processes within adipose tissue and increases the amount of non-esterified fatty acids (NEFA) in the bloodstream and some other tissues, while simultaneously decreasing their oxidation in skeletal muscle and the liver. This hormone mainly plays a role in protein metabolism by amplification of their biosynthesis and lowering their degradation in the liver, skeletal muscle, and adipose tissue [5].

## 3. What Does It Mean to Be Insulin Resistant?

Insulin resistance is clinically defined as a reduced response of target tissues to stimulation by insulin. The phenomenon of IR is accompanied by a pathological insulin secretion after a meal, which is called hyperinsulinemia. Interestingly, long-lasting hyperinsulinemia leads to aggravated IR. In line with that notion, a chronically elevated insulin level (e.g., due to improper insulin injections) produces an adaptive reduction in the number of plasma membrane receptors for the hormone (due to their adaptive internalization and degradation) [6]. Consequently, greater insulin dosage is required to elicit the same physiological effect, hence IR begins. Moreover, secondary alterations in target tissues are also possible. Marban et al. demonstrated that transgenic mice over-expressing insulin showed diminished insulin responsiveness despite fasting normoglycaemia and proper body weight [7]. This could be explained by an impaired binding of insulin to its receptors and/or stem from hypertriglyceridemia, which may impair insulin signal transduction [8]. Hyperinsulinemia may also promote weight gain, since insulin overdose results in severe hypoglycaemia and polyphagia (excessive eating) [9]. This leads to the formation of a specific vicious circle, i.e., hyperinsulinemia propels IR and weight gain, which, in turn, require higher insulin dosage for compensation. Moreover, also defects in the insulin receptor structure, a reduction in the density of the receptors at the cell surface, or improper action of the immune system, may lead to an abnormal response of target tissues to insulin.

**Figure 1:**
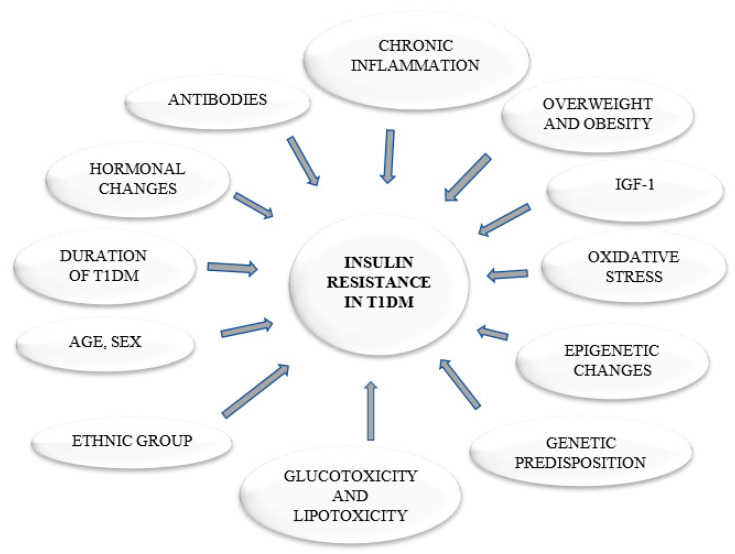
The causes of insulin resistance in type 1 diabetes.

The possibility of the existence of reduced tissue insulin sensitivity in children, adolescents, and adults that are diagnosed with T1DM is no longer put into question. The development of IR in T1DM may be triggered by many risk factors (presented in Figure 1). A key role in the initiation of IR in T1DM is attributed to the complex interactions between the genetic predisposition of an individual and their lifestyle. The human leukocyte antigen (HLA) accounts for most of the aforementioned genetic susceptibility to IR in T1DM. A study conducted by Todd et al. suggests that only the amino acid in position 57 of the DQB3-chain is strongly correlated with IR in T1DM [10]. IR is often associated with being overweight and/or obese. Therefore, priority has been given to non-pharmacological approaches that can inhibit the development of obesity in T1DM. Excessive adipose tissue mass leads to the development of IR by the overproduction of hormones antagonistic to insulin (growth hormone, glucagon, cortisol, and catecholamines), and through a direct secretion of increased amount of free fatty acids (FFA) into the blood. In the case of exorbitant FFA level, the body begins to use them as an energy source instead of glucose. Consequently, glucose is not underoxidized in the tissues and, thus, its level in the blood increases. Subsequently, insulin secretion is increased in order to maintain a normal blood glucose level. Moreover, chronically elevated FFA level in the blood may lead to their accumulation in peripheral tissues, where they may interfere with insulin signal transduction [11]. This is especially true for skeletal muscle, i.e., a tissue accountable for even 80% of insulin stimulated glucose uptake that takes place after a meal [12]. Under physiological conditions, insulin binds to its receptor and initiates the embedding of glucose transporters type 4 (GLUT-4) into the myocyte plasma membrane. The process is guided by a cascade of secondary signal transducers, the most important of which are: IRS-1, PI3K, PKB/Akt, and AS160 [13,14]. An excessive intracellular lipid build-up, especially into bioactive diacylglycerol (DAG) and ceramide (CER) species, disrupts this pathway. DAG, for instance, is known to activate protein kinase C (e.g., PKC θ) that inactivates IRS-1 (insulin receptor substrate 1). Ceramide, on the other hand, disables both IRS-1 (via activation of JNK) and protein kinase B (PKB/Akt) (via its proxy PP2A) [13,14]. Interestingly, Conway et al., in the Pittsburgh Epidemiology of Diabetes Complications Study, showed that, amongst adults with T1DM, the prevalence of overweight and obese individuals increased from 29% to 42%, and from 3% to 23%, respectively [15]. The participants were first seen in 1986–1988 (mean age and DM duration, were 29 and 20 years, respectively), and then after 18 years. Conway et al. suggested that the predictors of weight gain were: higher baseline hemoglobin A1c (HbA1c) concentration (directly related), symptomatic autonomic neuropathy (inversely related), overt nephropathy (inversely related), and intensive insulin therapy during follow-up (directly related) [15]. Fellinger et al. showed that patients with T1DM have higher BMI values when compared to the general population of Austria. However, the higher BMI values were observed for a group of the T1DM patients aged 30–49. This may be due to the fact that, in this age group, insulin therapy was conducted from the beginning of the diagnosis of T1DM. The higher BMI values were not related to 10.2% poorer glycemic level, which was examined by HbA1c [16]. Table 2 outlines a summary of the mechanisms that are involved in the etiopathogenesis of IR related to obesity. Unsurprisingly, it seems that, with worse metabolic stability of diabetes, IR severity also increases. The toxic effects of hyperglycemia and some lipid fractions also favor an increase in the resistance of insulin-dependent tissues to the action of insulin, thus enhancing IR and its metabolic consequences.

Oxidative stress interferes with insulin signaling, since reactive oxygen species (ROS) may induce insulin receptor substrate (IRS) serine/threonine phosphorylation, impair cellular redistribution of insulin signaling components, reduce *GLUT4* gene transcription, or alter mitochondrial activity [19]. Mechanisms, such as oxidative stress that is associated with hyperglycemia, lipotoxicity, and glucotoxicity are also responsible for the development of IR in T1DM. Oxidative stress causes an increase in the production of pro-inflammatory cytokines and, thus, the induction of inflammatory processes. In T1DM immune cells, such as macrophages, which are the source of pro-inflammatory cytokines, migrate to pancreatic islet cells. Chronic inflammation can also be included among the factors that contribute to the development of IR in T1DM. Adipose cell enlargement leads to a pro-inflammatory state and the formation of pro-inflammatory compounds, such as interleukin-6, C-reactive protein, and plasminogen activator inhibitor-1, which, in turn, contribute to the death of β-cells, modulation of β-cell regeneration processes, and IR [20,21]. Studies conducted by Gunawardana and Piston [22,23] seem to be in line with that notion. The authors investigated therapeutical effects of embryonic BAT (brown adipose tissue) transplants on the reversal of STZ- (streptozotocin) and autoimmune-mediated T1DM. The team was able to improve whole body glucose metabolism in mice. They attributed the success to the post-transplant increased the level of plasma insulin-like growth factor-I (IGF-I), a hormone with well-known anti-inflammatory properties [22,24].

The development of IR is also related to the gender of T1DM patients. Millstein et al. found that T1DM affected adipose and skeletal muscle insulin sensitivity to a greater extent in women than in men [25]. Participants in the control groups presented higher Morbus values (M-values) than those with DM, regardless of gender. Interestingly, women had a higher M-value than men from the respective control groups, whereas there were no differences by sex in M-values among the participants with T1DM. This result may suggest a greater deficit in the whole-body insulin sensitivity in women than in men with T1DM. Additionally, during the third stage of the glucose uptake study, some differences were observed for the values between women with and without T1DM [25]. During the first and second stages of the hyperinsulinemic-euglycemic clamp, the difference in FFA concentration by T1DM status was greater in women than in men during the least-squares means stages. These changes in IR in skeletal muscle and adipose tissue may be associated with disturbances in the hypothalamic-pituitary-gonadal axis in T1DM [25,26].

The severity of IR is variable and it depends on the influence of many factors. It is also related to the duration of T1DM [27,28,29,30]. A family history of T2DM has also been shown to be associated with greater IR in T1DM [27,31,32]. IR, as measured by the estimated glucose disposal rate (eGDR), is greater in racial/ethnic minorities as non-Hispanic blacks, Hispanics than in non-Hispanic whites [33,34].

A decreased insulin response is reported in adolescence when hormonal changes are observed, mainly in the growth hormone and sex hormones levels. It is often said that growth hormone has an anti-insulin effect because it impairs the ability of insulin to stimulate glucose uptake in peripheral tissues and enhances hepatic glucose synthesis. Growth hormone secretion is pulsatile throughout the day, but almost 50% of the daily secretion of this hormone occurs during the night. The dawn phenomenon happens as a result of increased blood glucose levels in the early morning hours (between 3 and 5 am) and it results in a significant increase in glucose levels on awakening. Physiologically speaking, between these hours the insulin level drops, which results in unblocking growth hormone secretion. A healthy person has a compensating mechanism in the form of additional insulin release, in people with DM this mechanism is disturbed, which leads to the appearance of pathology in the form of the dawn phenomenon. This event is most often observed in patients with T1DM, especially in children in their puberty period. It is related to the increased secretion of growth hormone by the pituitary gland during the night [35].

However, in adults, growth hormone deficiency may occur. It manifests itself by increased visceral obesity, IR, dyslipidemia, and hyperglycemia, and it contributes to increased cardiovascular morbidity and mortality. Insulin-like growth factor (IGF-1), which, like insulin, reduces blood glucose level, has anti-inflammatory properties, and it is important for the regulation of glucose uptake by peripheral tissues [36].

Growth hormone therapy has an antagonistic effect on insulin with respect to peripheral tissues, such as the liver, skeletal muscle, and adipose tissue. It increases glucose uptake by skeletal muscle and the liver and reduces it in adipose tissue. To compensate for the increase in circulating glucose level, following the administration of growth hormone, insulin production increases. As a result of growth hormone supplementation, an increase in lipolysis in visceral adipose tissue is observed, which leads to an increased number of FFA in the blood, which, in turn, causes the disruption of insulin signaling pathway and directly exerts toxic effect on β-cells [36].

Increased IR may be due to the presence of autoantibodies, i.e., antibodies against endogenous insulin and/or antibodies that are directed against the insulin receptor [37]. In case of some patients undergoing insulin therapy, the antibodies are produced against exogenous insulin [38,39]. This situation increases the risk of allergic reactions, large fluctuations in blood glucose levels, and increased insulin requirement.

IR severity/presence can be estimated by the: oral glucose tolerance test, the HOMA method for calculating the IR index (HOMA-IR), and the hyperinsulinemic-euglycemic clamp methods.

## 4. Quaternary Prevention

A Belgian general practitioner, Marc Jamoulle, coined the concept of quaternary prevention in 1995 [40]. According to the Wonca International Dictionary for General/Family Practice Quaternary Prevention was defined as: ‘action taken to identify a patient at risk of overmedicalization, to protect him from new medical invasion, and to suggest to him interventions, which are ethically acceptable’ [41]. The concept of quaternary prevention reflects more traditional levels of preventive medicine: primordial, primary, secondary, and tertiary preventions [42], as described below.

### 4.1. Primordial Prevention

Primordial prevention strategy encompasses all of the activities that aim to limit the occurrence of unhealthy behaviors and promote the correct ones. Primordial prevention includes actions targeted to whole society. Currently, there is no way to delay T1DM, but a reduction in the risk of IR development in T1DM is plausible [43]. Therefore, early risk reduction plays a key role in prophylaxis of the IR occurrence in T1DM. In 1989, at the forty-second World Health Assembly, WHO presented a health promotion proclamation and the introduction of multi-level coordinated DM prevention, which is supported by the International Diabetes Federation (IDF) [44]. This proclamation calls on the Member States to keep reliable statistics on the epidemiological status of DM. The declaration provides a support for the Member States and their activities targeted at the prevention of DM and its complications [44]. This is important for planning and implementing preventive measures, tailored to the needs of local communities, which aimed to counteract and monitor the development of IR.

A very important element of primordial prevention is the implementation of a coordinated international policy in health education, trade, agriculture, transport, and urban planning; this would enable the members of a society to make healthy choices and learn healthy habits. Healthy choices can be promoted in schools, workplaces, and homes, which will have an impact on the health of society [45]. This can also be achieved by the decision regarding the amount of tax that is imposed on food products, including sweets, sweetened drinks, as well as alcohol and cigarettes. It is also worth emphasizing that the prices of vegetables, fruit, and fish should be set so that the products are more accessible to residents [46]. Modern lifestyle is characterized by a lack of physical activity and a sedentary lifestyle. The IDF recommends that a person stays physically active three to five times a week for at least 30–45 min [47]. Therefore, during urban planning, a sufficient number of playgrounds, open-air gyms, and bicycle paths should be ensured.

IR is not a direct cause of T1DM, but rather an accompanying phenomenon. Nevertheless, it is a burden, since people with this type of DM and concomitant IR will need higher insulin doses in order to keep their blood glucose level stable (as compared to the people with DM, but without IR). The availability and price of insulin is currently a major concern for patients, their families, healthcare professionals, insurers, and employers. Between 2007 and 2016, as prices rose and more expensive insulin products were introduced, the average total Medicare Part D spending per insulin user increased by 358%, from $862 to $3949. In the same years, the out-of-pocket expenses on insulin increased from $236 million to $968 million. This was due to both the increase in insulin prices and number of users. An average per capita insulin spending increased from $324 to $588, which is an increase of 81% [48]. In 2018, the American Diabetes Association conducted a survey [49]. The results indicated that 25% of people with DM rationed their insulin stock, because they could not afford to take the whole prescribed amount. Among the respondents, 23% reported the need to change the insulin to a cheaper one or a different brand. Interestingly, 23% and 20% of the patient submitted to take 1–2 required doses of insulin per week or month, respectively. Worryingly, 36% of the respondents were forced to choose between purchasing a medicament or paying for other medical services. On account of the type of insurance, 22% of the respondents had to change insulin due to its price [48,49].

The problem of rising insulin prices is quite complex and it is influenced by many factors. Only three companies control 90% of the global insulin market (Eli Lilly, Novo Nordisk, and Sanofi). Often, only one of these companies supplies insulin in a given country, which permits them to dictate the prices of their products. The key factor behind the price of insulin is the existence of patents that give companies a monopoly on specific inventions, usually for up to 20 years. Previous-generation insulin, as well as older animal-derived insulins, are currently excluded from the patent. However, pharmaceutical companies use loopholes in the patent system to patent other elements that are related to a basic drug. This decreases the competition on the market and keeps insulin prices high [48]. The above is of particular importance for the uninsured and the high-split insured individuals. If current trends are maintained, the annual cost of insulin treatment could reach $121.2 billion by 2024. This suggests a necessity to change the current pricing practices [48].

It turns out that a device that almost every inhabitant of the globe has can play an important role within the primordial prevention framework. In 2013, the WHO, in collaboration with the International Telecommunications Union, introduced the mDiabetes service on a large scale. The utility was originally intended to help countries, such as Senegal. The initiative Be He@lthy, Be Mobile was aimed at designing, implementing, and expanding services for the prevention and treatment of DM and a range of non-communicable diseases [50]. Through SMSs, their recipients had easier access to information regarding the disease and health education. This allows for a reduction in morbidity and treatment costs and lets the patients live longer and in better health. Senegal was the first country, which, in 2014, launched a targeted mDiabetes campaign to help people manage fasting during Ramadan. In 2016, the application was launched in India, where it currently supports over 96,000 users. The WHO also conducts annual campaigns in Egypt. In 2017, the campaign reached over 175,000 people worldwide [50]. As Dr. Douglas Bettcher, director of the WHO’s NCD Prevention Department, emphasizes, it is important to provide information to the public in a form that is both simple and action-oriented. That way, it is easier to incorporate the recommendations into a person’s daily activities and make positive changes to their diet, exercise, and habits. It is also very important for patients to be able to take care of their health during time periods between visits to the doctor or health care professional. This is essential for improving the quality of life and treatment outcomes [50].

### 4.2. Primary Prevention

Primary prevention aims to avert the occurrence of a specific disease by reducing the risk of the disease by changing people’s behaviors or their exposure to risk factors that in turn may lead to the disease development. This type of prophylaxis is aimed at a specific endangered group of people. At this level of prevention, activities at the local stage play a very important role, where we can see cooperation and intermingling of activities of local governments with non-governmental organizations (NGOs) [51]. There are programs in the field of counteracting alcoholism and tobacco smoking. Workshops are often held in order to learn to cook healthier versions of familiar foods. In the line with the IDF recommendations for a healthy diet for the general society, individuals are endorsed to replace fruit juices, soda, and other sugar-sweetened beverages with water, tea, or coffee. Additionally, they are urged to eat at least three servings of raw vegetables a day and fruit up to three servings a day. Suggestions are also put forward to choose lean meat, fish and seafood, whole wheat bread, and unsaturated fats. The above is often supported by classes that teach how to read the labels on food products.

The Trial to Reduce Insulin Dependent Diabetes Mellitus (IDDM) in the Genetically at Risk Study (TRIGR), which is an international, randomized, and double-blinded trial, is an interesting example of primary prevention. The research was conducted in 78 study centers from 15 countries, between May 2002 and January 2007, and follow-up continued until the youngest participant reached 10 years of age in February 2017. A total of 2159 newborn infants who had a first-degree relative with T1DM and defined human leukocyte antigen (HLA) genotypes were recruited to this research [52]. The study aimed to test “whether hydrolyzed infant formula compared to cow’s milk-based formula decreases the risk of developing T1DM in children with increased genetic susceptibility”. The TRIGR Study Group found that weaning to the hydrolyzed formula did not reduce the risk of T1DM in children with increased disease risk. The finding indicates that there is no need for revision of the dietary recommendations for a newborn at risk for T1DM [53].

Pacaud et al., in a secondary analysis of the TRIGR study, compared the clinical characteristics and development of β-cell autoantibodies in patients with a family history of T1DM. The research involved 2074 children from families with a single family member (mother, father, or sibling) affected by T1DM [54]. The study showed that the risk to develop β-cell autoimmunity was significantly lower (*p* < 0.001) in children with maternal T1DM than with other family members with T1DM. This indicates that more attention should be paid to the other genetic and epigenetic risk factors or the immunological mechanisms. That approach should allow for better identifying those at risk of developing T1DM and to better plan future prevention strategies.

Other findings from the TRIGR primary prevention study include that early postnatal vitamin D may confer protection against the development of T1DM [55]. Krischer et al. also showed evidence of the relationships between atopic eczema, allergic rhinitis, or persistent asthma and diabetes-related autoimmunity. Research proved that, for eczema, the interaction depends upon the appearance of the autoantibody [56].

### 4.3. Secondary Prevention

The aim of secondary prevention is the early detection and treatment of pathological changes while maintaining control over the development of the disease. Screening tests are the first step in implementing early interventions, which, when considering the condition of economy, are much more profitable than later treatment. Once disease screening tests detect a disease that is in the acute clinical phase, secondary prevention aims to improve the patient’s quality of life.

These activities can be carried out at the level of a family doctor, which, in Poland, is often additionally scored by the National Health Fund. The family doctor can order the examination of blood pressure, urine, sugar level, lipid profile, and BMI. These tests belong to the foundation of basic tests ordered by a general practitioner and, although cheap, are often sufficient for predicting the risk of developing IR in a patient with T1DM.

A good example of secondary prevention could be research that was conducted by TrialNet, which is the largest clinical trial network for T1DM [57]. The Teplizumab Prevention Study was the first study conducted in humans in order to show that clinical T1DM could be delayed for two years in children and adults with a high risk of developing the disease [58]. The study was conducted from 2011 to 2018 in the United States, Canada, Australia, and Germany. The research was conducted among nondiabetic relatives of patients with T1DM; they were at least eight years of age and at a high risk of developing clinical DM. Each participant received a 14-day treatment with teplizumab or saline, administered intravenously. Although the research results are very promising, they have some limitations. The study was based on a very small cohort group, 76 people, relatives of people with T1DM, and non-Hispanic white participants. Future research should involve not only the relatives of people with T1DM, but also those who are at risk of developing DM. Different ethnic groups should be also included. The participants of this study only received one cycle of teplizumab administration. Perhaps multiple administration would provide additional benefits and exert its effect on more people. Nevertheless, the research that was conducted by TrialNet is a step to achieving their goal of “a future without T1DM” [57].

### 4.4. Tertiary Prevention

Tertiary prevention with its activities aims to stop the progression of the disease and reduce its complications. These activities should take place at the outpatient level. This requires the cooperation of the patient, doctors, and specialists in many fields, like a diabetologist, a medical rehabilitation physician, a neurologist or an ophthalmologist, and a dietitian and physiotherapist [59]. Patients are often referred to specialized sanatorium centers and, after their return, they are still under the care of specialists. Tertiary prevention is focused on preventing further adverse effects of IR, including disability. The rehabilitation aims to restore the lost functions. The health education of a patient and his relatives is a very important element that may help the patient to cope with their limitations and prevent secondary social isolation that can lead to secondary disability and the formation of a vicious circle [60]. Health education shall focus on teaching how to: (a) self-monitor blood glucose level, (b) determine the appropriate time for blood glucose measurement, (c) eat healthy, and (d) properly inject insulin. The health education training should be repeated.

Another important element of health education while using of the appropriate language concerning people with T1DM. Proper language habits help to avoid stigmatization, not only of people with T1DM, but also of their relatives and closest social environment. Dickinson et al. paid special attention to the language and gave recommendations in order to facilitate cooperation with people with DM. The language, which we use, should be respectful, person-oriented, and devoid of stigmatization. The publication provides recommendations consistent with the guidelines of the American Psychological Association and should be used by diabetologists, diabetes educators, researchers, or those who deal with people with diabetes in their environment [61]. Language has power and it can significantly affect our perception or behavior. Dunning et al. also emphasized that the way we talk to the people with diabetes and about them is of great importance and can help improve their involvement in the process of diabetes treatment. Moreover the language may better treatment outcomes, and has a positive impact on an individual psycho-social well-being [62]. Future diabetes prevention programs should include educational classes that will teach the language that will not stigmatize a person with T1DM. LaManna et al. also emphasized the importance of health education as an important resource that helps to reduce the risk and incidence of hypoglycemia and improve the quality of life of people with T1DM [63].

## 5. Treatment

Metformin is a drug of choice in T2DM due to its safety and efficacy, as well as its multi-metabolic effects. Not only does it lower glycemic stability by sensitizing tissues to the impact of insulin, but it also increases glucose uptake by adipose tissue and supports the process of FFA re-esterification. Through these processes, it prevents lipolysis and the release of FFA to the blood. This drug increases the activity of lipoprotein lipase, lowers the concentration of triacylglycerol, as well as total and LDL cholesterol. It also has anti-inflammatory and antioxidant properties.

Many studies have been conducted on the appropriateness of introducing metformin to the pharmacotherapy of patients with T1DM. The results indicated that these patients obtained an improvement in total insulin dose, basal insulin dose, a modest reduction in weight, or lipids level (total and LDL cholesterol), but only during short term observation [64,65,66,67]. Some studies also confirmed that metformin may lower HbA1c level [65,66]. The above-mentioned positive effects are no longer observed with long-term drug supplementation. During many years of research, no significant changes in body weight, total daily insulin dose, basal insulin dose, improvement in lipids level, or fasting plasma glucose or HbA1c level were observed in the patients [67,68,69,70]. Some studies also indicated the possible side effects of metformin application, such as an increased risk of adverse gastrointestinal effects [64,69,71,72]. Some studies also report vitamin B12 deficiency in patients with T1DM [66,72]. For patients with T1DM, this vitamin is important due to the reduction of the high homocysteine level, which is considered to be a major risk factor for the formation of atherosclerotic plaque. Vitamin B12 also participates in the formation of myelin sheaths of neurons, which are necessary for the process of nerve impulses conduction [73]. Its deficiency can lead to nerve damage.

Metformin is successfully used for the treatment of people with T2DM; however, not much is known regarding its impact on T1DM. A longitudinal study conducted by Staels et al. that encompassed 10 years timespan proved the lack of long-term beneficial effects of metformin therapy on weight loss, HbA1c level reduction, or diminishing insulin dosage requirements. Therefore, this drug is not expected to bring satisfactory effects, even after a long-term pharmacotherapy of patients with T1DM [68].

An ideal drug should meet several criteria, as Drzeworski emphasized. First of all, it should reduce IR in T1DM and protect β-cells, and, thus, reduce the need for insulin. Secondly, it should also reduce hyperglycemia, body weight, oxidative stress, and the risk of cardiovascular complications that stem from many years of its administration [74]. Important features of the ideal drug also include: its affordable price, high safety, improvement of the patient’s life quality, and extension of its duration. Although metformin meets many of these criteria, future studies in search for an ideal medication for IR in T1DM are still warranted [75].

However, we should remember the words of the doctor of medicine, the court physician of Polish kings, Wojciech Oczko, who often repeated that “Movement can replace almost any medicine, but all medicines taken together can’t replace movement”.

## 6. Conclusions

The existence of reduced tissue insulin sensitivity in children, adolescents, and adults diagnosed in T1DM is no longer being questioned. Our present analysis, which focused on IR in T1DM, presented that many factors influence the development of this disorder. A better understanding of the risk factors for the development of IR in T1DM is still needed in order to improve quaternary prevention. Research has showed that the inclusion of prophylaxis may prevent IR in T1DM or reduce its negative effects. Probably, with respect to primary and secondary prevention of IR in T1DM, it would not matter if the actions that were carried out under primordial prevention were more effective and covered a larger number of the world’s populations. To be effective, prevention requires commitment and organized action at all levels, from the activities of international organizations to the involvement of people with T1DM.

## Figures and Tables

**Table 1:** Comparison of type 1 and type 2 diabetes mellitus.

Feature	T1DM	T2DM
body weight at the time of diagnosis	within the normal range or underweight	overweight or obese
cause of the disease	insulin deficiency as a result of β-cells damage	insulin resistance
presence of antibodies	anti-GAD, ICA, IA2, IAA, ZnT8	not found
disease onset	acute with accompanying diabetic ketoacidosis	mild onset
basic pharmacotherapy	insulin	initially metformin and other orally administered medications, in some cases insulin
Does the occurrence of the disease depend on a patient’s lifestyle?	no	yes

anti-GAD: anti-glutamic acid decarboxylase; ICA: islet cell antibodies; IA2: islet antigen 2; IAA: insulin autoantibodies; ZnT8: zinc transporter 8.

**Table 2:** The mechanisms involved in the etiopathogenesis of insulin resistance related to obesity [17,18].

Pre-Receptor	Receptor	Post-Receptor
↓ access of insulin to muscle secondary to free fatty acids excess	insulin receptor downregulation secondary to hyperinsulinemia	inhibition of the intracellular cascades by several adiposity-related factors (e.g., ↑free fatty acids, impaired adipokines, and/or cytokines secretion)
abnormal hormone structure	↓ affinity of the receptor for the hormone	glucose transporter abnormalities
the presence of insulin binding antibodies		
insulin degradation		
the presence of insulin antagonists such as glucagon, cortisol, thyroid hormones		
